# A Case of Recurrent Multiple Myeloma as Testicular Plasmacytoma Without Systemic Disease

**DOI:** 10.7759/cureus.40785

**Published:** 2023-06-22

**Authors:** Krishna Doshi, Sabree Abedrabo, Jacob Bitran, Nahren Asado

**Affiliations:** 1 Internal Medicine, Advocate Lutheran General Hospital, Park Ridge, USA; 2 Hematology and Oncology, Advocate Lutheran General Hospital, Park Ridge, USA; 3 Pathology, Advocate Lutheran General Hospital, Park Ridge, USA

**Keywords:** plasma cell neoplasms, extramedullary plasmacytoma (emp), testicular plasmacytoma, relapsing multiple myeloma, extramedullary multiple myeloma

## Abstract

Plasma cell neoplasms include various conditions ranging from indolent conditions such as monoclonal gammopathy of undetermined significance (MGUS) to more aggressive forms such as multiple myeloma (MM). The World Health Organization classifies plasmacytomas into two types: solitary osseous plasmacytoma (SOP) and extramedullary plasmacytoma (EMP). Most primary EMPs occur in the upper gastrointestinal tract, head and neck, upper respiratory system, central nervous system, lungs, liver, spleen, and kidneys. However, the occurrence of EMP involving the testis site is quite rare. Given the rarity of testicular plasmacytoma, there is no consensus on the standard of treatment for this diagnosis. Most EMP is radiosensitive, with few localized types responding to surgical intervention. Tumor recurrence and disseminated infiltration are treated with adjuvant chemotherapy after radiation or surgery. Our patient has a unique presentation of an individual who developed recurrent myeloma of the testis 12 years after his initial diagnosis of myeloma.

## Introduction

Plasmacytoma is defined as a discrete mass of neoplastic monoclonal plasma cells occurring in the bone or at an extramedullary site [1]. Common sites of occurrence are usually the head and neck, upper respiratory system, gastrointestinal tract, and central nervous system [2]. Testicular plasmacytoma, whether as a primary source or a reflection of underlying multiple myeloma (MM), is a rare manifestation [3]. Testicular plasmacytoma has an estimated incidence between 0.03% and 0.1% [4,5]; in the context of MM alone, it has an incidence between 0.6% and 2.7% [4]. Given its rare occurrence, we report an interesting case of a patient who presented with recurrence 12 years after diagnosis of MM.

## Case presentation

The is an 81-year-old male diagnosed with IgG lambda MGUS in 2003, 20 years before his current presentation. Plasma cell assessment at the time of diagnosis demonstrated IgG lambda 0.4 g/dL with a normal kappa to lambda ratio. The patient was being followed every six months, and in 2010, his M protein began to rise to 1.4 g/dL with a slightly elevated kappa to lambda ratio of 2.98. Bone marrow biopsy was recommended; however, the patient was lost to follow-up.

In March 2015, the patient presented with worsening back pain and anemia, with hemoglobin dropping from a baseline of 14.5-10.7 g/dL. Repeat serum protein electrophoresis demonstrated an M protein of 1.53 g/dL. Bone marrow biopsy revealed increased cellularity at 45% with increased 20% plasma cells consistent with progression to MM. Cytogenetic noted 45, X, −Y/46 on chromosome 15, XY. FISH analysis performed on the same specimen was positive for trisomy 11, monosomy 13, and trisomy 17 in 6-7% of cells. PET-CT demonstrated FDA avid T12 vertebral lesion. Beta-2 microglobulin was normal at 2.1 mg/L. However, albumin was low at 2.4 g/dL; therefore, the patient thus was reviewed for international stage II. He was then treated with three cycles of cyclophosphamide, bortezomib, and dexamethasone (CyBorD) and was recommended for an autologous bone marrow transplant; however, the patient declined.

Less than a year later, in February 2016, surveillance laboratories showed early relapsing myeloma with rising M protein from undetectable to 0.9 g/dL and an elevated kappa to lambda ratio of 38.75. He was then treated with four cycles of carfilzomib and dexamethasone and once again was recommended for an autologous bone marrow transplant for consolidation. He agreed, and in September 2016, he underwent a stem cell transplant with a high dose of melphalan conditioning regimen.

In 2019, the patient would once again develop early relapse disease with an elevated M protein of 0.3 g/dL and elevated kappa to lambda ratio of 10.0. Repeat bone marrow biopsy demonstrated 10-15% plasma cell of bone marrow. Repeat FISH and cytogenetics were identical to those at the time of progression to MM in 2015. He was treated with CD38 monoclonal antibody daratumumab and achieved complete remission. He was placed on monthly maintenance of daratumumab.

**Figure 1:**
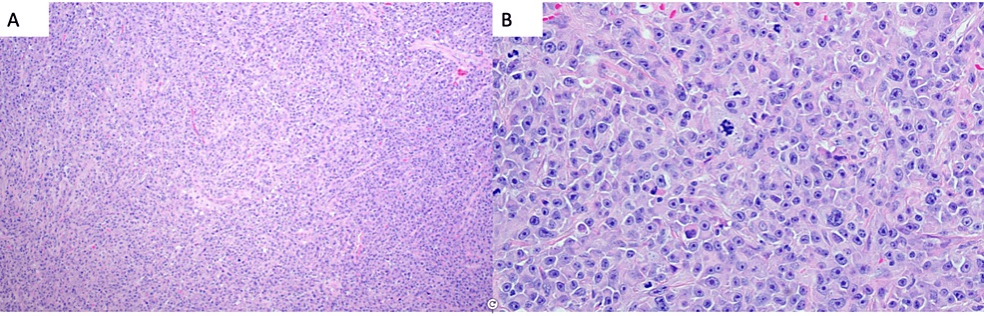
(A) Population of tumor cells infiltrated as diffuse sheets. (B) Atypical plasma cells with plasmablastic morphology, atypical mitoses, and macronucleoli

**Figure 2:**
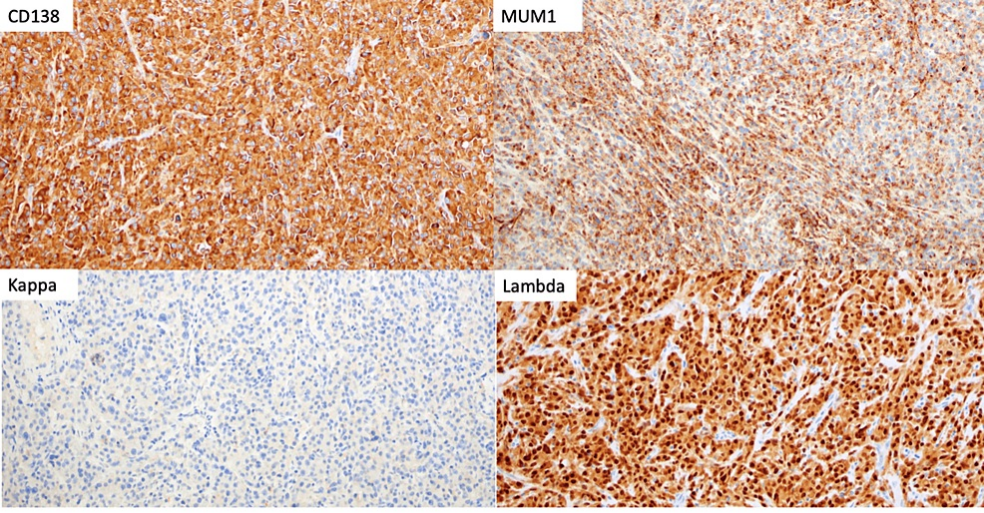
Immunohistochemical staining showing diffuse and strong positivity for CD138, MUM1, and lambda while negative for kappa

**Figure 3:**
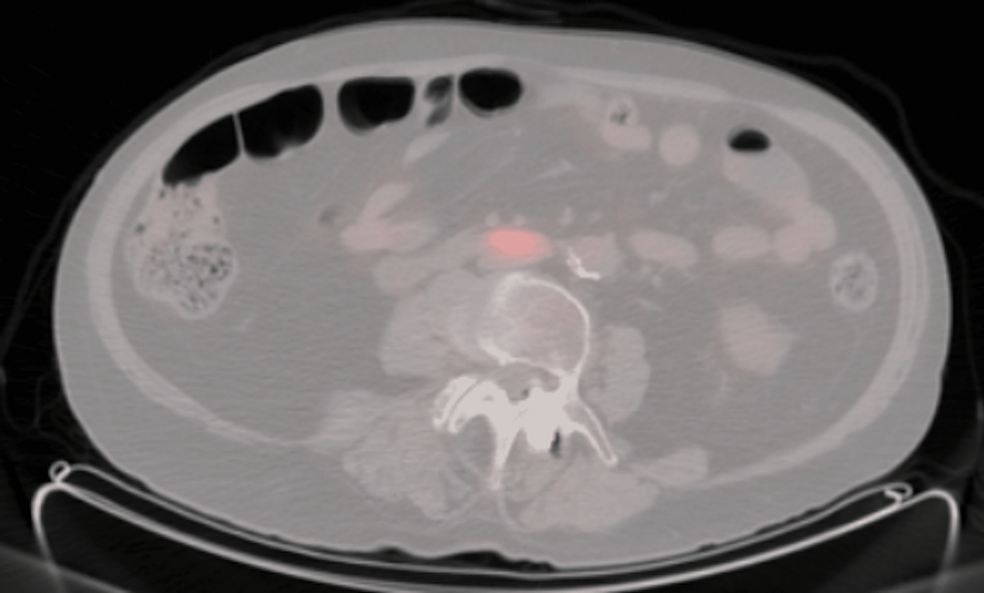
PET-CT staging

In February 2023, he noticed a right testicular mass that was demonstrated on the testicular ultrasound. At this time, the patient remained in remission with an M protein of 0.1 g/dL. In March 2023, the patient underwent right orchiectomy, and pathology demonstrated that testis with involvement by plasma cell neoplasm with MUM1, CD138, and lambda stains are diffusely strongly positive while kappa, AE1/AE3, and CD20 are negative (Figures 1, 2). PET-CT staging demonstrated two FDG avid lymph nodes in the abdomen along the great vessels, suspicious for metastatic lymphadenopathy (Figure 3).

## Discussion

Plasma cell neoplasms comprise a spectrum of diseases ranging from monoclonal gammopathy of undetermined significance (MGUS) to a more aggressive MM and plasma cell leukemia [6]. The WHO recognizes two types of plasmacytoma: solitary osseous plasmacytoma (SOP) and extramedullary plasmacytoma (EMP) [6]. Common locations of occurrence are the head and neck, gastrointestinal tract, upper respiratory system, and central nervous system [7]. Testicular plasmacytoma is highly uncommon and estimated to account for 0.03-0.1% of all testicular malignancies or 2% of all plasma cell neoplasms [7,8]. One study noted that the median age of patients was 51 years and higher than that of testicular cancer [9]. It was also reported that the majority of the patients demonstrated previous or simultaneous occurrences of MM and plasmacytoma [9].

The mean age of patients diagnosed with testicular plasmacytoma is 55-60 years [1]. Usually, patients present with a testicular mass that may be tender. Sonographic imaging has shown that they are hypoechoic and can be homogenous or heterogenous [1]. The tumor demonstrates sheets of plasma cells, on microscopy, with varying degrees of differentiation. Ultimately, immunostaining the tissue sample confirms the diagnosis with the presence of CD138, CD79a, and light chain restriction [10]. Our patient’s tumor mass stained positive for CD138.

EMP usually responds well to local radiotherapy. For testicular plasmacytoma, orchiectomy, followed by chemotherapy or radiotherapy, EMP is considered a therapeutic option [9-11]. One possible hypothesis for recurrence in the testicular site is the lack of penetration of bortezomib through the blood-testis barrier, which makes the testis a more likely site for disease recurrence. The current literature reports that several newer chemotherapeutic approaches have been used to treat EMP. Lenalidomide and dexamethasone have shown positive results in managing patients with relapsed or refractory MM [11]. Of the 18 cases reported in the study, 44% showed complete disappearance of the EMP [11]. The hypothesis of the mechanism of action is that lenalidomide may have an antiproliferative effect on myeloma cells irrespective of its existence in bone marrow stroma or other tissue microenvironments [12]. Additionally, lenalidomide has a potent anti-tumor activity through anti-angiogenic effects, and prior bortezomib treatment does not affect its response [9]. Calvo-Villas et al. noted that three out of 11 patients demonstrated re-lapse; thus, newer strategies were needed to overcome treatment resistance of the disease [11]. More data have been reported regarding the use of lenalidomide as maintenance therapy for patients with MM.

## Conclusions

Overall, EMPs in the testis are rare. It is even rarer to have testicular plasmacytoma in the absence of systemic MM. We presented a unique case of a patient with recurrent MM with testicular plasmacytoma 12 years after his initial diagnosis. We hope this will add to the current literature and aid clinicians in considering it a differential diagnosis in patients who do not have systemic or biochemical MM.
